# Isolation of fresh endothelial cells from porcine heart for cardiovascular studies: a new fast protocol suitable for genomic, transcriptomic and cell biology studies

**DOI:** 10.1186/s12860-019-0215-2

**Published:** 2019-08-13

**Authors:** Olli-Pekka A. Hätinen, Johanna E. Lähteenvuo, Henna J. Korpela, Juho J. Pajula, Seppo Ylä-Herttuala

**Affiliations:** 0000 0001 0726 2490 grid.9668.1 A.I. Virtanen Institute for molecular sciences, University of Eastern Finland, Yliopistonranta 1E, 70211 Kuopio, Finland

**Keywords:** Porcine heart, Endothelial cell, Isolation, Fresh tissue, Myocardial, Pig

## Abstract

**Background:**

Endothelial cells (ECs) play a key role in tissue homeostasis, in several pathological conditions, and specifically in the control of vascular functions. ECs are frequently used as in vitro model systems for cardiovascular studies and vascular biology. The porcine model is commonly used in human clinical cardiovascular studies. Currently, however, there is no robust protocol for the isolation of porcine heart ECs. We have developed a fast isolation protocol, which is cost effective, takes only 1–2 h, and produces EC purity of over 97%. This protocol is optimized for porcine hearts but can be adapted for use with other large animals.

**Methods:**

Heart is washed by flushing with PBS, whereafter endothelial cells are detached by collagenase incubation and the cells can then be collected immediately after the incubation and plated within an hour after the heart is isolated from a pig.

**Results:**

The swiftness of the protocol limits changes in the phenotype and RNA expression profile of the cells. Cells were identified as ECs with CD31 (PECAM-1) antibody immunostaining. Functionality of ECs were ensured with in vitro angiogenesis assay. The purity of the ECs was verified by using fluorescence assisted cell sorting (FACS) with the CD31 antibody.

**Conclusion:**

We developed a new, fast, and cost-effective isolation method for pig heart ECs. Successful isolation of pure ECs is a prerequisite for several cardiovascular and vascular biology studies.

## Background

Endothelial cells (ECs) play a key role in the maintenance of vascular functions. Human umbilical vein endothelial cells (HUVECs) have served as a model system for many studies of the etiology of vascular diseases and the regulation of vascular homeostasis. These studies have provided significant knowledge about the functions of ECs and the development of vascular and malignant diseases. However, the phenotype and the functionality of ECs vary in different tissues [1, 2]. Because of these specific differences, it is recommended to use ECs from the specific tissue in question. For that reason, ECs from heart are required for studies on myocardial diseases.

**Fig. 1:**
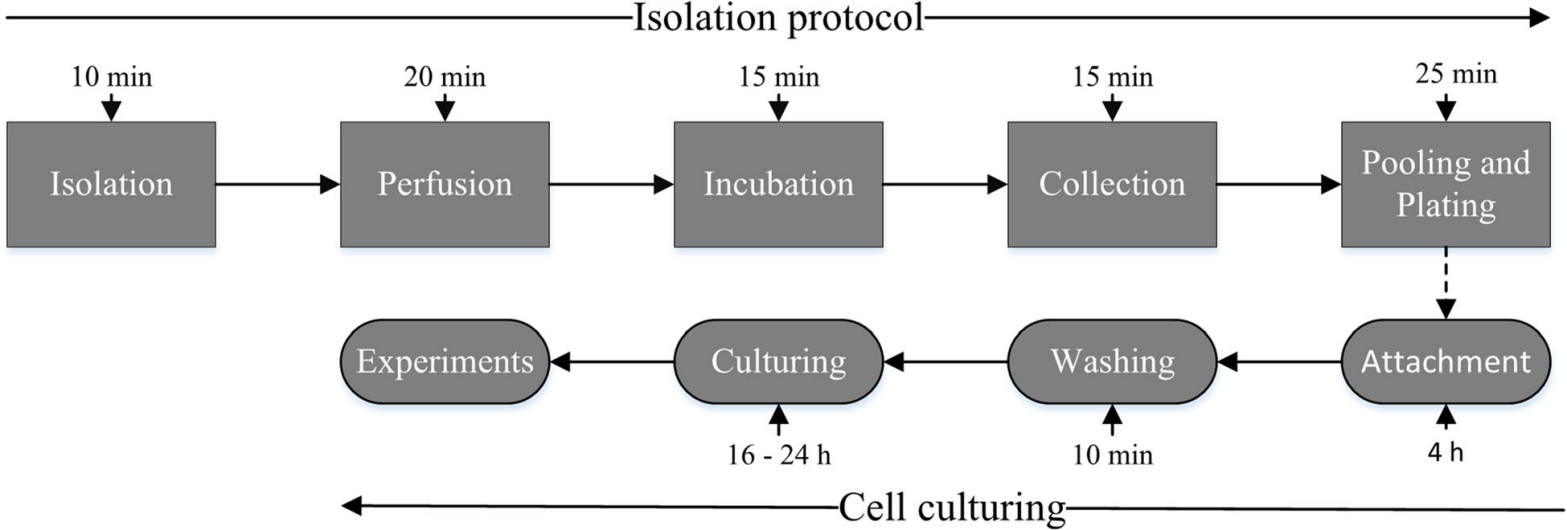
Timeline of endothelial cell isolation protocol. Isolation protocol takes around 1–2 h to complete depending on the skill level of a scientist. Isolation protocol includes: isolation of the heart, perfusion (setuping heart for perfusion, PBS perfusion and perfusion of Collagenase solution), Incubation (37 °C water bath), collection of endothelial cells, pooling and plating (includes time needed for sentrifucation, removal of supernatant, pooling cells and plating them on fibronectin gelating-coated plates). Cell culturing includes: attachment of endothelial cells, washing and culturing time. Cells can be used for experiments or passaged when plates are 80–90% confluent

Pigs are the most frequently used large animal for cardiovascular studies because of its similarities to human. However, to our best knowledge, no fast protocol for the isolation of pig heart ECs exists. Current isolation protocols are based on mechanically mincing and enzymatically digesting the heart tissue to a cell suspension, which is not feasible for a pig heart due to its large size [3]. Consequently, the mouse heart is usually used as the source of heart ECs. Even in the mouse heart cell suspension, most of the cells are fibroblasts and cardiomyocytes. Therefore, ECs must be labeled with antibodies to isolate them from the cell suspension. The labeling is expensive and time-consuming taking usually at least 4 h [3]. Such protocols cause changes in EC transcription and phenotype [4–7], potentially changing tissue specific properties. To retain these specific properties, we developed a fast protocol to isolate ECs from porcine heart. The protocol takes only 1–2 h to complete (Fig. 1). In addition, the use of commercially available equipment allows this protocol to be adapted for use with other large animals [8].

## Results

### Pre-medication and sacrification

Five ml of Heparin (B. Braun, 2500 I.U./ml) was found to be critical for perfusion process. As an anticoagulant, Heparin makes removal of the blood easier. Intra-vascular (IV) injection of potassium chloride (KCl) was used to arrest the heart in diastole, the phase in which the heart muscle is relaxed and veins are easier to perfuse.

### Isolation protocol

The whole protocol is performed under sterile conditions and at room temperature (RT). However, all the solutions were preheated to normal pig body temperature (37 °C) to ensure best conditions for the cells. The whole protocol takes a maximum of 1–2 h to get the cells on the cell culture plates. The protocol provides approximately 2 × 10^5^ to 3 × 10^5^ endothelial cells from each heart. The swiftness of the protocol limits changes in the phenotype and RNA expression profile of the cells.

### Identification of endothelial cells

Cells were identified as ECs with CD31 (PECAM-1) antibody (MCA1746GA, AbD Serotec) (Fig. 3 A) immunostaining. Tube formation is one of the key characteristics of ECs [9] and functionality of ECs were ensured with in vitro angiogenesis assay (Matrigel™ GFR Membrane Matrix, #356231, Corning, USA) (Fig. 3 B). Tube formation were observed in three different time points (p2, p3 and p5). The purity of the ECs was verified by using fluorescence assisted cell sorting (FACS) (Fig. 3 C). The CD31 antibody (MCA1746GA, AbD Serotec, USA) was used for the FACS assay.

### Endothelial cell culturing

ECs were passaged maximum of 13 times, however, after passage 6 there were visual indications of the change in phenotype. There were no observed differences between cells isolated from individual pigs.

## Discussion

The quickly growing field of cell and tissue studies requires more and more specific types of primary cells from various organs. Pig is the most frequently used large animal for cardiovascular studies because of its similarities to human. Therefore, we have focused on the isolation of pig heart endothelial cells instead of other species. There is no published protocol for quickly isolating pig heart ECs. Known methods are used mainly for mouse studies and are based on mechanically mincing and enzymatically digesting the heart tissue to a cell suspension. It is crucial that the isolation method is fast so it retains the specific properties ECs.

The main challenge in the protocol is EC recovery which may vary depending on how well cells are detached from each other. Occasionally, cells clog up inside the vessels obstructing the flow of the liquids, leaving many cells in the clogged vessels. This can be visually detected as the clogged region of the heart becomes heavily swollen. The exact reason for clogging remains unclear. However, when this does not happen, the isolation procedure provides large numbers of pure ECs, which are ready to be used for genomic, transcriptomic and cell biology experiments.

Perfusion of only coronary arteries is challenging. Perfusion of the whole heart makes it possible that some of ECs could detach from leaflets of aortic valve or endocardium of the ventricles. However, we have established that shear stress in the coronary arteries lead to detachment of the ECs after the collagenase incubation. The pressure and flow rate on the ventricles are much lower than in the coronary arteries as the aortic valve is closed. In addition, concentration of collagenase will be decreased if any of it has flown in to the ventricles. In the future studies, it would be interesting to characterize the ventricular endocardial and leaflets endothelial cells as well.

The protocol can also be adapted for other large animals because of the equipment used are commercially available. The successful isolation of ECs is an essential source of cell cultures to be used for numerous studies, and therefore, it could also reduce the number of animal experiments.

## Conclusion

We have developed a step-by-step method to isolate ECs from porcine heart. This protocol is a fast and inexpensive way to isolate ECs and offers new possibilities for the emerging fields of genomic, transcriptomic, cell and tissue studies in cardiovascular medicine and vascular biology.

## Methods

### Isolation method

All animal procedures were approved by The National Animal Experimental Board of Finland and carried out in accordance with the guidelines of The Finnish Act on Animal Experimentation. For this study, healthy, female pigs (*n* = 6; Finnish Landrace-Yorkshire; Emolandia Oy, Finland) weighting 20–25 kg were used. After arrival at the FELASA accredited laboratory animal (The National Laboratory Animal Center of The University of Eastern Finland), animals were housed at stalls specifically intended for pigs. Clinical examination of the health status were performed at the arrival and on daily basis. Animals were kept in standard housing conditions. Diet and water were provided ad libitum.

Animals were randomly selected for the cell isolation. Pigs were pre-medicated with combination of atropine (0.05 mg/kg im; Atropin, Takeda GmbH, Austria) and azeperone (8 mg/kg im; Stersnil, Elanco, USA). General anaesthesia were induced with propofol (5 mg/kg iv bolus; Propolipid, Fresenius Kabi, Germany). Pigs were pre-medicated with 5 ml of Heparin (B. Braun, 2500 I.U./ml). A single animal was used for each cell isolation. The isolation of heart and ECs were performed under sterile conditions. Pigs were sacrificed with an IV injection of potassium chloride (KCl) and heart (approximately 250–350 g) was harvested and transferred to the fume hood at RT.

**Fig. 2:**
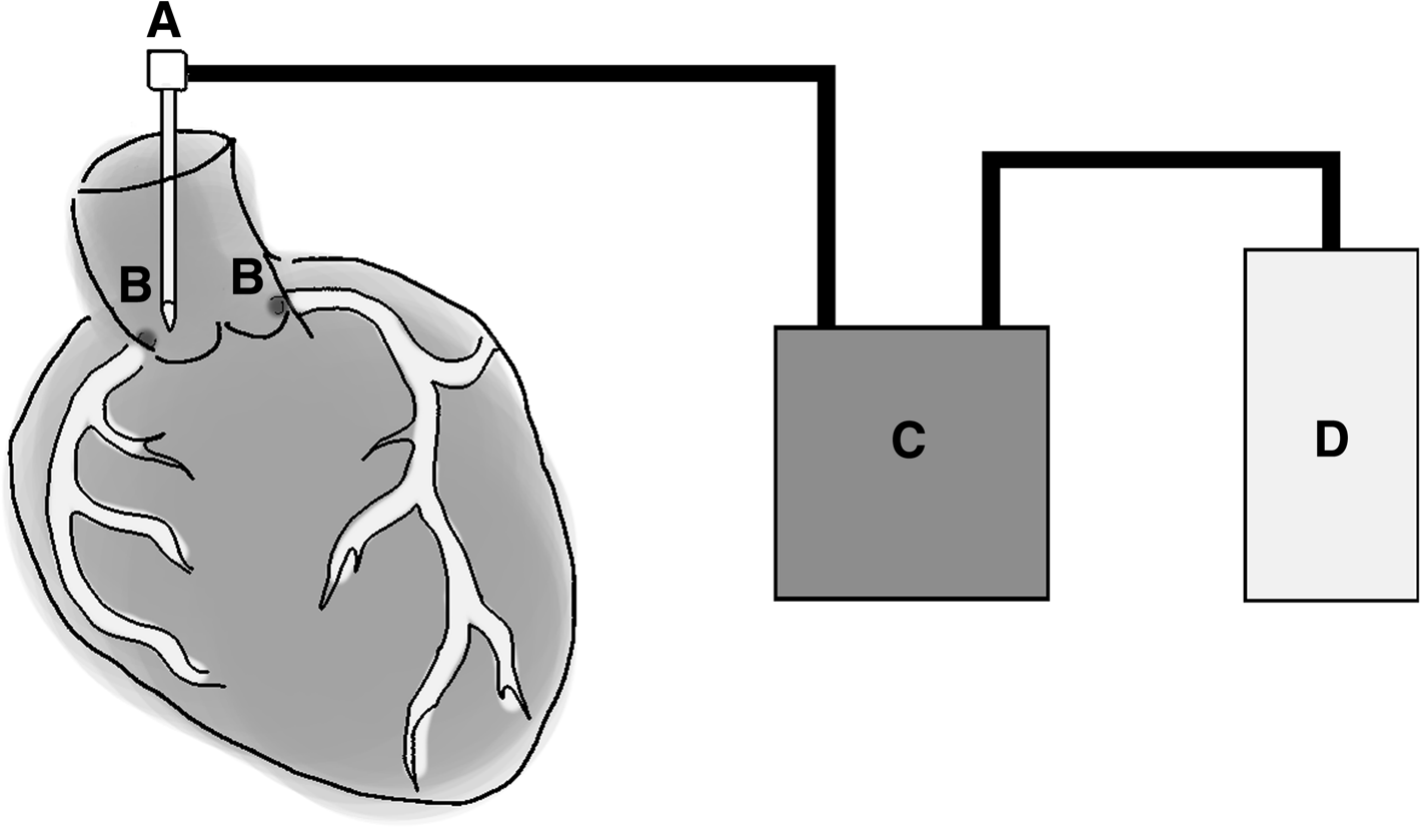
Illustration of the endothelial cell isolation system. Firstly, the needle (**a**) placement is critical for a successful isolation. It should be located right above the openings of the coronary arteries (**b**) in aortic arch. A Masterflex L/S infusion pump (**c**) is started with a medium speed (400 ml/min) to wash and fill the ventricles with PBS (**d**). After the ventricles are washed, the pump is stopped so that the aortic valves will be closed. The pump is restarted after 30 s with a low speed (100 ml/min) so that flowing liquid (PBS) will not push the valves open but flows into the coronaries instead. Aorta is closed with medical clamps. After that, you should feel pressure on the aortic arch and coronary flow should start. The perfusion is continued until the flushing liquid is clear. Image courtesy of Dr. Henna J. Korpela

Heart was perfused using a Masterflex L/S infusion pump (Model 77,200–62, Masterflex, IL, USA) with a 19G needle attached to a Masterflex L/S 24 tube (Masterflex, IL, USA). The needle was carefully pushed into the aortic arch just above coronary arteries (Fig. 2). Aorta, pulmonary arteries and other blood vessels were clamped to provide pressure for liquid to flow into the coronary arteries. One to two litres of PBS (Dulbecco’s Phosphate Buffered Saline, D8537, Sigma-Aldrich, USA) was used for perfusion to remove blood from arteries, veins and ventricles.

**Fig. 3:**
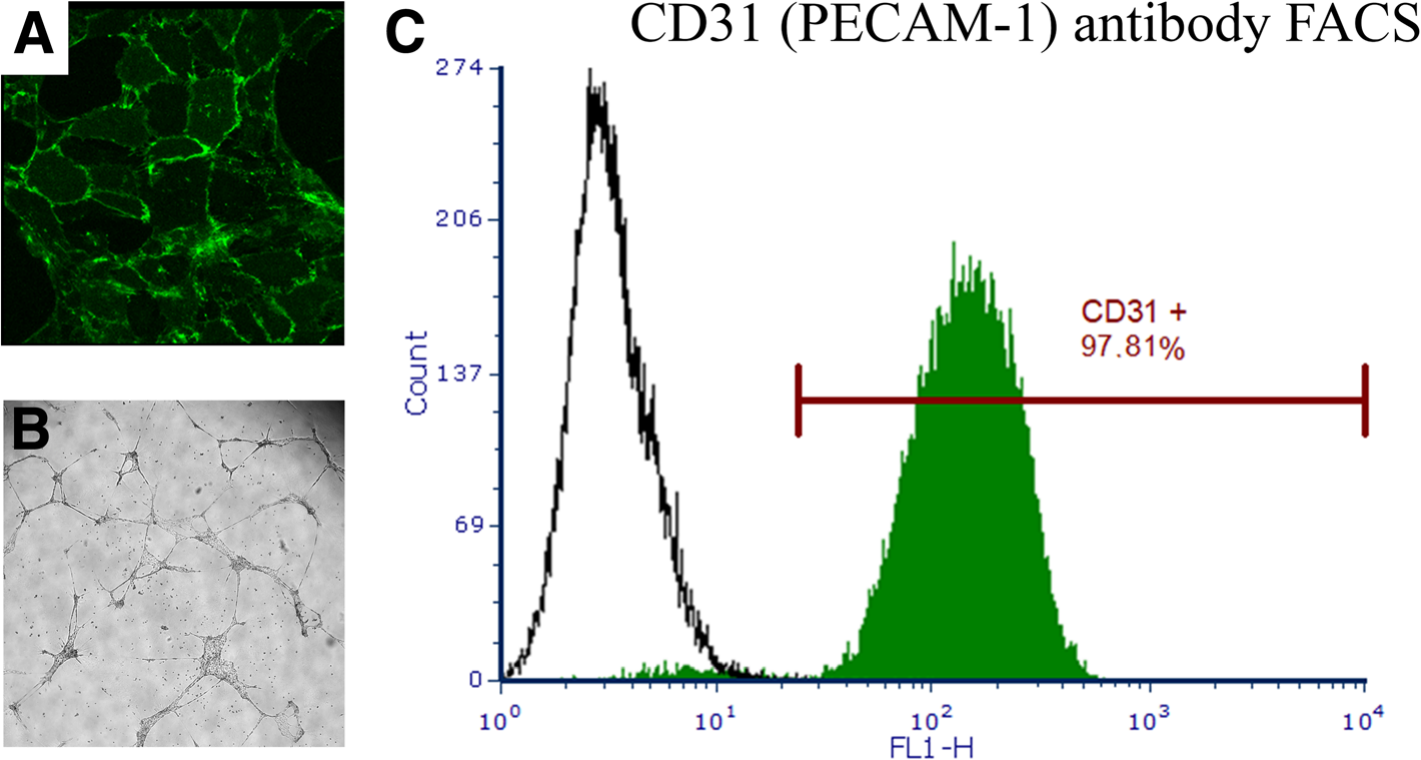
Identification of functionality and purity of endothelial cells. **a**) Isolated cells were identified as endothelial cells by CD31 (PECAM-1) antibody (MCA1746GA, AbD Serotec) immunostaining (passage 3). **b**) One of the characteristics of endothelial cells is tube formation. Functional analysis of the cells was performed by using an angiogenesis assay (Matrigel™ GFR Membrane Matrix, #356231, Corning) (passage 2). **c**) Purity of the cells was ensured by using fluorescence assisted cell sorting (FACS) resulting in purity of 97.81%, which can be considered as a pure preparation of ECs (passage 2)

Next, the detaching solution (approximately 250 ml, 37 **°**C, 0.3 mg/ml [37.5 CDU/ml]) of Collagenase II [C6885, Sigma-Aldrich, USA] and IV [C5138, Sigma-Aldrich, USA]) was perfused into the vascular system of the heart and incubated for 15 min. After the incubation, the heart was perfused with 800 ml of PBS and the overflowing liquid containing ECs was collected into 50 ml tubes. Tubes were centrifuged (700 xg, 4 °C for 15 min) and the supernatants were removed. Pellets were pooled by using 10 ml of Complete Medium (1000 ml Dulbecco’s Modified Eagle’s Medium [Gibco, USA]; 4.5 g/ml glucose; 200 ml 20% FBS [Gibco, USA]; 0.5 ml 1 M HEPES pH 7.4 [Gibco, USA]; 10 ml penicillin/streptomycin [Gibco, USA]; 5 ml 100 mM 1x Non-Essential Amino acids [Gibco, USA]; 5 ml 100 mM 1X Sodim Pyruvat [Gibco, USA]; 2.5 ml 1 mM L-glutamine [Gibco, USA]; 5 mg Endothelial mitogen [Biomedical Technologies Inc., USA]; 2 ml 500 UI Heparin [B. Braun, Germany]). Plates were coated with 5 ml of fibronectin (10 μg/ml, #F2006, Sigma, USA)-gelatin (0.5 mg/ml, #G-6144, Sigma, USA) in PBS and incubated at 5% CO_2_, 37 °C for 30 min. Excess fibronectin-gelatin was removed after the incubation. Cells were plated on fibronectin gelatin-coated 100 mm cell-cultured plates. The plates were kept at 5% CO_2_, 37 °C for 4 h to let ECs to attach on the plates. The plates were then washed with 3 × 10 ml of PBS to remove the remaining red blood cells. After the washing, 10 ml of Complete Medium was added to the cells. The medium was changed 16–24 h after the washing. Cells were passaged when the plates were 80 to 90% confluent. The isolation procedure provides large numbers of pure ECs (Fig. 3), which are ready to be used for genomic, transcriptomic and cell biology experiments.

### Immunostaining

Endothelial cells (passage 3) were plated on Nunc™ Lab-Tek™ II Chamber Slide™ (154,523, Thermo Fisher, USA). Cell permeabilization was achieved by 15 min incubation with 0.1% Triton X-100 in PBS (600 μl/well). Primary antibody (200 μl/well, dilution 1:50, CD31 (PECAM-1) [MCA1746GA, AbD Serotec]) was incubated for 60 min at RT. Wells were incubated 15 min with 600 μl of PBS, 0.1% Triton X-100 in PBS and again with PBS. Secondary antibody Alexa Fluor 488 (200 μl/ml, dilution 1:200, A-21202, Thermo Fisher, USA) was incubated for 60 min at RT. Wells were washed again again with PBS, 0.1% Triton X-100 in PBS and PBS for 15 min and then stored with 600 μl of PBS. Immunostainings with no primary antibody incubation were used as controls.

### Fluorescence assisted cell sorting (FACS)

At passage 3, ECs were detached with trypsin and transferred into 15 ml centrifuge tubes. Cells were centrifuged 600 xg for 6 min. Supernatants wa removed and cells were resuspended with 1 ml of 2% PFA-PEM in PBS and incubated for 30 min at RT. After incubation, tubes were centrifuged 1000 xg for 3 min. Supernatant was removed and cells were washed with PBS and centrifuged as above. 500 μl of blocking serum (10% FBS in PBS) was added to tubes and incubated for 30 min at RT. Tubes were centrifuged as above and supernatant removed. Cells were incubated 60 min at RT with primary antibody (200 μl of 0.5% FBS in PBS and 1 μl of CD31 [MCA1746GA, AbD Serotec]). Tubes were centrifuged as earlier and supernatant removed. Cells were washed twice with PBS. 200 μl of secondary antibody Alexa Fluor 488 (dilution 1:200, A-21202, Thermo Fisher, USA) was incubated for 60 min at RT. Cells were centrifuged and washed as above. Cells were suspended with 750 μl of PBS and analyzed with the fluorescence-activated cell sorter (FACSCalibur, Becton Dickinson, USA). Immunostaining, negative control and primary antibody control were included in the analyses.

### Tube formation

Matrigel (Matrigel™ GFR Membrane Matrix, #356231, Corning, USA) was thawn overnight at 4 **°**C. 0.289 ml per well of Matrigel (10 mg/ml) was pipetted with cooled pipet heads on a cooled 24-well cell-cultured plates. The plate was incubated at 5% CO_2_, 37 °C for 60 min. The remaining liquid was carefully removed.

Cells were detached from 80 to 90% confluent 100 mm cell-cultured plates with 1 ml of trypsin (1 min at 5% CO_2_, 37 °C). Effect of trypsin was stopped by 5 ml of Complete Medium and cells were collected into 15 ml tubes. The amount of cells was calculated from cell suspension. Cell suspension was diluted to concentration of 500.000 cells/ml. 300 μl of diluted cell suspension was pipetted per well. Cells were incubated at 5% CO_2_, 37 °C for 16 h.

After the incubation, remaining liquid was removed from the wells. Cells were washed with PBS and fixed for 15 min with 300 μl of 1% Glutaraldehyde-2% PFA in PBS solution. Fixation solution was removed and storage solution (PBS) was added. Imaging of the wells was done with confocal microscope (Axio Observer Z1, Zeiss, Germany).

## Data Availability

The datasets used and/or analysed during the current study are available from the corresponding author on reasonable request.

## Abbreviations

CDU: Collagen Digestion Unit
EC: Endothelial cell
FACS: Fluorescence assisted cell sorting
HUVEC: Human umbilical vein endothelial cell
PFA-PEM: cytoskeleton-protective buffer
RT: Room temperature
